# Supplementary material for: Two Rare Variants in PLAU and BACE1 Genes—Do They Contribute to Semantic Dementia Clinical Phenotype?
Source: Genes (Basel). 2021 Nov 17;12(11):1806. doi: 10.3390/genes12111806 (PMC8624613; doi:10.3390/genes12111806)
Supplement: Supplementary file 1 [file genes-12-01806-s001.zip › genes-1438972 spplementary materials.pdf]

APPENDIX A

Supplementary files

Supplementary Table S3: Control fibroblast lines used for the study

Control line	Gender/age of donation	Health status	reference
Control 1 (C1)	F/65	Neurologically healthy	[1,2]
Control 2 (C2)	M/69		
Control 3 (C3)	F/56		

Supplementary Table S4: Primers used in the study

Gene	Primer sequences	source
GAPDH	GTTCGACAGTCAGCCGCATC<br>GGAATTTGCCATGGGTGGA	[3,4]
PLAU	CCAAAGAAATTCGGAGGGCA<br>CTTGGACAAGCAGCTTTAGGC	Designed for this study (NCBI primer blast)
DSCR1	GCACCAGCTCAAGAAGGAACC<br>GGGACTCAAATTTGGCCCTGG	[5]
BACE1<br>*(exon 6F, exon 8R)*	CACCACCAACCTTCGTTTGC<br>AAGCCAATTCGTTTTCGGGC	Designed for this study (NCBI primer blast)
BACE1<br>*(exon 3F, exon 9R)*	CAACTGGGAAGGCATCCTGG<br>GCAGGGAGATGTCATCAGCA	Designed for this study (NCBI primer blast)

Supplementary Table S5. The results of the neuropsychological assessment conducted in the proband's brother at the age of 63 when he presented with restricted behavioral repertoire and marked cognitive rigidity.

<table>
<tr><th>Function assessed / test</th><th>Score</th><th>Comments</th></tr>
<tr><td colspan="3">General cognitive status</td></tr>
<tr><td>ACE-III: orientation and attention / 18</td><td>17</td><td rowspan="5">clearly semantic errors in confrontation naming: penguin was named as "a bird"

rhinoceros was named as "wild boar"</td></tr>
<tr><td>ACE-III: memory / 26</td><td>22</td></tr>
<tr><td>ACE-III: fluency / 14</td><td>14</td></tr>
<tr><td>ACE-III: language / 26</td><td>24</td></tr>
<tr><td>ACE-III: visuospatial function / 16</td><td>16</td></tr>
<tr><td>ACE-III total score / 100</td><td>93</td><td>within normal range</td></tr>
<tr><td>Mini-ACE (MACE) total score / 30</td><td>24</td><td>below cut-off</td></tr>
<tr><td colspan="3">Language function & visual / verbal semantics</td></tr>
<tr><td>Naming
BNT / 30
ACE-III / 12</td><td>
19
10</td><td>mild anomia</td></tr>
<tr><td>Verbal fluency (raw scores / 60s.)
- semantic: animals
- fruit & vegetables / fish
phonemic: K / P / S</td><td>
22
16 / 12
18 / 19 / 11</td><td>semantic fluency slightly lower than expected in the context of good phonemic fluency; in fish trial the patient started with aquarium fish in which he has special interest;

two incidents of other personal comments during assessment (referring to the patient's dog)</td></tr>
<tr><td>Semantics
Sydney Language Battery (SYDBAT):
- Naming (% correct)
- Word Comprehension (% correct)
- Semantic Association (% correct)
PALS – animal naming / comprehension
- object naming / comprehension
Recognition of famous faces* / 15
Naming famous faces*: spontaneous / cued
Drawings: to command / copy
- guitar:
- anchor
- lightbulb
- snail
- rhinoceros</td><td>

19 (63%)
20 (66%)
21 (70%)
9 / 10
8/10
14
7 / 14

both correct
incorrect / correct
simplified / correct</td><td>naming, word comprehension and semantic association are similarly impaired in SYDBAT which indicates a semantic deficit

in PALS "eagle" was named "flying dinosaur" (after the patient correctly named dinosaur)

the rhinoceros (drawing to command) lacked any distinctive features, it was drawn as a four-legged domestic animal, resembling a sheep;

anchor (drawing to command) – only the bottom part was drawn</td></tr>
</table>

	both correct<br>incorrect / correct	
**Language Comprehension**<br>Commands from BDAE / 15	15	preserved
**Repetition**<br>Sentence repetition from BDAE<br>high frequency / low frequency (max. 8)	8 / 8	preserved
**Reading**<br>Word reading from BDAE / 30<br>Sentence reading from BDAE / 10	30<br>9	one verb distortion in sentence reading
**Writing**<br>Primer level dictation from BDAE / 15<br>Writing words to dictation from BDAE / 10<br>Written confrontation naming / 10<br>Written picture description (beach scene)	15<br>10<br>10<br>qualitative	writing to dictation was preserved (the testing did not address surface dysgraphia)<br>three characters in the beach scene were named "man" (also the boy building a sand castle), the girl were misnamed as a boy
**Other aspects of cognition**		
**Visuospatial function**<br>VOSP: Incomplete letters / 20<br>Silhouettes / 30<br>Dot counting /10<br>Position discrimination / 20<br>Number location / 10<br>Cube analysis / 10<br>CFT copy / 36	20<br>8<br>9<br>20<br>10<br>10<br>30	mostly preserved;<br>the only visual subtest that was failed was the one with semantic involvement (Silhouettes)
**Working memory**<br>Digit span – max. forward / backward<br>Corsi – max. span forward<br>TMT A<br>TMT B	4 / 4<br>5<br>80 sec.; 60T<br>129 sec.; 52T ; 0 errors	mostly preserved;<br>verbal immediate memory was slightly deficient
**Episodic memory**<br>Address recall from ACE-III:<br>- learning curve / delayed recall (max. 7)<br>- recognition (max. 5)	6-5-7 / 5<br>5	prose delayed recall was relatively well preserved, but logical error (likely due to a semantic deficit) appeared (the patient recalled that the load was heavier as salt dissolved);<br>face delayed recognition was well preserved;

10-word list learning:<br>- learning curve<br>- delayed recall<br>Recognition: correct / false positives<br>CFT – after 3 min. delay<br>CFT after 30 min. delay<br>CFT recognition<br>Famous faces after a delay:<br>- free recall<br>- cued recall<br>- recognition<br>Story recall* (max. 10):<br>- story 1: immediate (free / cued / recognition)<br>- story 2: immediate (free / cued / recognition)<br>- story 1: delayed (free / cued / recognition)<br>- story 2: delayed (free / cued / recognition)	 3-4-4-7-7<br>5<br>9 / 4<br>31T<br>35T<br><20T<br><br>6<br>8<br>15<br><br>8 / 8 / 10<br>8 / 10 / -<br>7 / 7 / 10<br>9 / 9 / 9	word-list learning and figure recall were deficient
**Executive function**<br>Sorting Weigl blocks<br>Tower of London – DX-2: total correct SS<br>total move SS<br>rule violation SS	 8 / 12<br>98<br>116<br>64	poor inhibition
**Calculation**<br>Serial sevens (max. 5)<br>Calculation from WAB, multiple choice / 24<br>Written arithmetics<br>Arithmetic word-problem solving / 8	 4<br>24<br>few errors in complex tasks<br>4	calculation was slightly impaired (problems only in complex tasks)
**Praxis**<br>Alternate hand movements<br>Spatial praxis<br>Ideomotor praxis<br>Efferent motor praxis:<br>1st sequence / 2nd sequence (max. 5)	 correct<br>correct<br>correct<br><br>5/5	preserved

ACE-III- Addenbrooke's Cognitive Examination-III; BDAE- Boston diagnostic Aphasia Examination; BNT-Boston Naming Test; CFT- Complex Figure Test; PALS – Progressive Aphasia Language Scale; WAB- Western Aphasia Battery; TMT-Trail Making Test; VOSP- Visual Object and Space Perception Test

*famous face recognition (celebrity recall) and story recall tasks were adapted versions of tasks used in Manchester neuropsychological assessment battery (with permission of Prof. Julie Snowden), see [6]

Supplementary Table S6: The proband's results of Addenbrooke's Cognitive Examination-III (ACE-III) from the age of 69 till the age of 73.

	Age at assessment			
ACE-III	*69 y.o.*	*71 y.o.*	*72 y.o.*	*73 y.o.*
orientation and attention (max. 18)	15 (83%)	8 (44 %)	4 (22%)	1 (5%)
memory (max. 26)	7 (27%)	8 (31 %)	4 (15%)	0 (0%)
verbal fluency (max. 14)	9 (64%)	5 (36 %)	1 (7%)	0 (0%)
language (max. 26)	13 (50%)	12 (46 %)	9 (35%)	1 (4%)
visuospatial function (max. 16)	15 (94%)	12 (75%)	11 (69%)	7 (44%)
total score (max. 100)	59	45	29	9
M-ACE (max. 30)	17	7	4	0

Supplementary Table S7: The results of detailed neuropsychological assessment in the proband

	69 y.o.	*71 y.o.*	*72 y.o.*	*73 y.o.*
**Language function & visual / verbal semantics**				
**Naming**				
BNT / 30	2	0	-	-
ACE-III / 12	3	2	1	0
**Verbal fluency** (raw scores / 60s.)				
- semantic: animals / fruit	9 / 7	5 / 4	0 / 0	0 / -
fish / vegetables / clothes	1 / 8 / 13	0 / 0 / 10	0 / 0 / 2	-
- phonemic: K / P / S / M	17 / 23 / 24 / -	10 / 13 / 13 / -	3 / 4 / - / 3	0 / - / -/ -
**Language Comprehension**				
Commands from BDAE / 15	15	15	13	3
**Visual and verbal semantics**				
Pyramids and Palm tree test:	-	63%	42%	62%
- pictorial version (% correct)	-	71%	65%	58%
- verbal version (% correct)				
Sydney Language Battery:	30%	7%	0%	-
- Naming (% correct)	53%	37%	33%	3%
- Word Comprehension (% correct)	33%	37%	47%	-
- Semantic Association (% correct)				
**Repetition**				
Sentence repetition from BDAE				-
high frequency / low frequency (max. 8)	7 / 8	7 / 7	7/8	-
**Reading**				
Word reading from BDAE / 30	30	30	30	30
Sentence reading from BDAE / 10	10	10	10	10
**Writing**	-			
Primer level dictation from BDAE / 15		15	15	9
Writing words to dictation from BDAE / 10		10	10	4
Written confrontation naming / 10		9	5	-
**Other aspects of cognition**				
**Visuospatial function**				
VOSP: Incomplete letters / 20	19	19	9	-
Dot counting /10	10	9	10	9
Position discrimination / 20	20	20	-	-
Number location / 10	9	9	discontinued	-
Cube analysis / 10	8	9	7	5
JoLO / 30	-	-	22	-
CFT copy / 36	34	36	34	-
**Working memory**				
Digit span – max. forward / backward	5 / 3	7 / 4	6 / 3	discontinued
Corsi – max. span forward	-	5	-	5
TMT A	45s.; 57T	120s., >100T	139s., >100T	discontinued
TMT B	216s., 85T, 5 errors	234s., 89T, 0 errors	-	-
**Episodic memory**				
Address recall from ACE-III:				
- learning curve / delayed recall (max. 7)	5-6-7 / 0	4-4-6 / 0	3-4-4 / 0	0-1-0 / 0
- recognition (max. 5)	0	2	0	0
10-word list learning:				
- learning curve	3-5-6-6-7	5-6-3-5-6	5-3-3-3-4	-
- delayed recall	0	0	0	-
Recognition: correct / false positives	6 / 3	5 / 11	7/17	-
CFT – after 3 min. delay	<20T	<20T	-	-
CFT after 30 min. delay	<20T	-	-	-

CFT recognition	<20T	-	-	-
**Executive function**				
Sorting Weigl blocks	11 / 15*	7 / 9**	-	-
Tower of London – DX-2: total correct SS	-	98	-	-
total move SS	-	≤ 60	-	-
rule violation SS	-	≤ 60	-	-
**Calculation**				
Serial sevens (max. 14)	-	12	-	-
Calculation from WAB, multiple choice / 24	24	24	22	16***
**Motor function and praxis**				
9 Hole Peg test	-	-	35s. / 36s. (slowing due to compulsive tendencies)	-
Alternate hand movements	preserved	preserved	preserved	-
Spatial praxis	preserved	preserved	preserved	-
Ideomotor praxis	preserved	preserved	semantic errors	-
Efferent motor praxis:				
- 1st sequence / 2nd sequence (max. 5)	5/ 5	5 / 5	4 / 0	-

ACE-III- Addenbrooke's Cognitive Examination-III; BDAE- Boston diagnostic Aphasia Examination; BNT-Boston Naming Test; CFT- Complex Figure Test; WAB- Western Aphasia Battery; TMT-Trail Making Test; VOSP- Visual Object and Space Perception Test

*attempting 5 sorting criteria; ** attempting 3 sorting criteria; ***written calculation as the patient could not understand the multiple choice procedure

Supplementary Table S8. Human Splicing Finder Analysis of BACE1 wariant, Fig S4 C: Significant alteration of ESE / ESS motifs ratio according to Human Splice Finder Proffesional webtool (https://hsf.genomnis.com/home)[6]

Name	Position	Sequence
ESE_ASF (ESE Site Broken)	chr11:117291048	CGGAGAA
ESE_ASFB (ESE Site Broken)	chr11:117291048	CGGAGAA
PESE (ESE Site Broken)	chr11:117291048	CGGAGAAG
PESE (New ESE Site)	chr11:117291048	TGGAGAAG
RESCUE ESE (New ESE Site)	chr11:117291050	GATGGA
Sironi_motif2 (New ESS Site)	chr11:117291050	GATGGAG
Fas ESS (New ESS Site)	chr11:117291051	AGATGG
EIE (New ESE Site)	chr11:117291052	CAGATG
PESE (ESE Site Broken)	chr11:117291052	CAGACGGA
ESE_9G8 (ESE Site Broken)	chr11:117291053	ACAGAC
ESE_SRp40 (ESE Site Broken)	chr11:117291053	ACAGACG
ESE_ASF (New ESE Site)	chr11:117291054	CACAGAT
Sironi_motif1 (New ESS Site)	chr11:117291055	ACACAGAT

Signal - Alteration of auxiliary sequences	Interpretation - Significant alteration of ESE / ESS motifs ratio (-5)
Alteration of auxiliary sequences	Significant alteration of ESE / ESS motifs ratio (-5)

Supplementary Table S9. Human Splicing Finder Analysis of BACE1 wariant, Fig S4 C: Significant alteration of ESE / ESS motifs ratio according to Human Splice Finder Proffesional webtool (https://hsf.genomnis.com/home)[7]

Name	Position	Sequence
ESE_ASF (ESE Site Broken)	chr11:117291048	CGGAGAA
ESE_ASFB (ESE Site Broken)	chr11:117291048	CGGAGAA
PESE (ESE Site Broken)	chr11:117291048	CGGAGAAG
PESE (New ESE Site)	chr11:117291048	TGGAGAAG
RESCUE ESE (New ESE Site)	chr11:117291050	GATGGA
Sironi_motif2 (New ESS Site)	chr11:117291050	GATGGAG
Fas ESS (New ESS Site)	chr11:117291051	AGATGG
EIE (New ESE Site)	chr11:117291052	CAGATG
PESE (ESE Site Broken)	chr11:117291052	CAGACGGA
ESE_9G8 (ESE Site Broken)	chr11:117291053	ACAGAC
ESE_SRp40 (ESE Site Broken)	chr11:117291053	ACAGACG
ESE_ASF (New ESE Site)	chr11:117291054	CACAGAT
Sironi_motif1 (New ESS Site)	chr11:117291055	ACACAGAT

Signal	Interpretation
Alteration of auxiliary sequences	Significant alteration of ESE / ESS motifs ratio (-5)

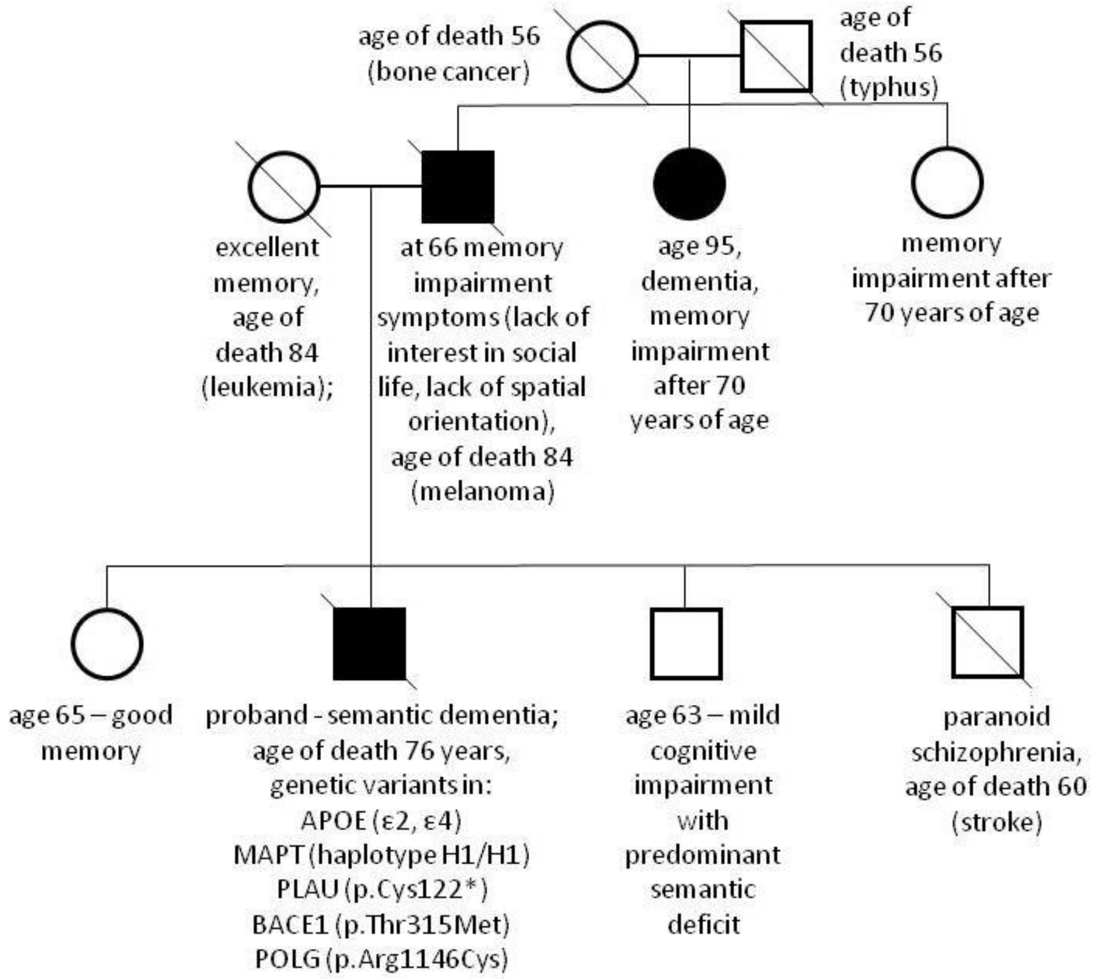

Supplementary Figure S1: Family history of the patient;

A) B)

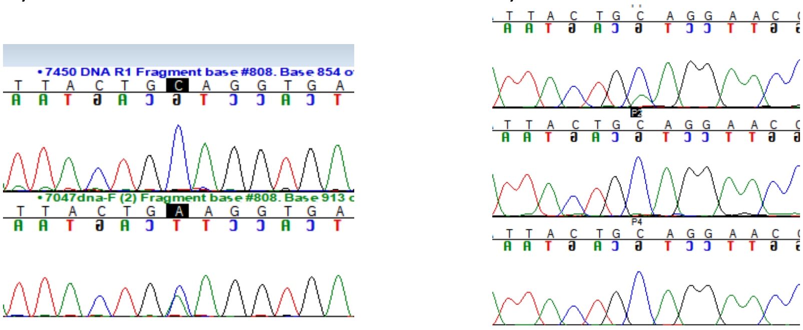

Supplementary Figure S2. Sanger sequencing of patient's DNA and cDNA, A) *PLAU* - DNA from control individual and the patient (upper and lower panel, respectively); B): *PLAU* – patient's cDNA after puromycine treatment showing a re-appeareance of the allele with the PLAU mutation (upper panel), cDNA without treatment from the patient (middle panel) and from control individual (lower panel);

A) B)

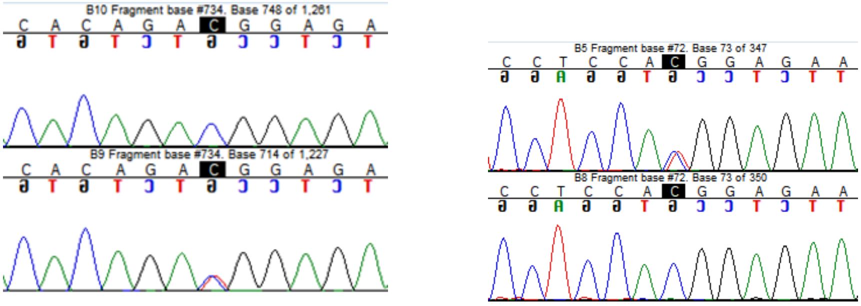

Supplementary Figure S3. Sanger sequencing of: A) *BACE1* - DNA from control individual and the patient (upper and lower panel, respectively); B) *BACE1* – patient's cDNA (upper panel); cDNA from the control individual (lower panel)

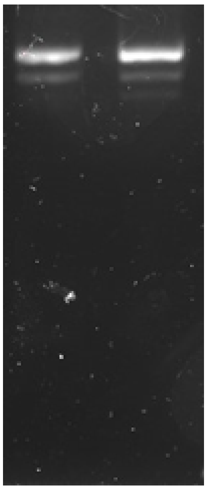

Supplementary Figure S4. Agarose gel electrophoresis of the PCR products for patient's cDNA and control's cDNA with BACE1 primers 3F-9R.

References:

1. Gaweda-Walerych, K.; Sitek, E.J.; Narozanska, E.; Wezyk, M.; Brockhuis, B.; Zekanowski, C.; Slawek, J. Functional characterization of a novel progranulin mutation in a patient with progressive nonfluent aphasia. *Neurobiol Aging* **2018**, *72*, 186 e189-186 e112,
2. Gaweda-Walerych, K.; Walerych, D.; Berdynski, M.; Buratti, E.; Zekanowski, C. Parkin Levels Decrease in Fibroblasts With Progranulin (PGRN) Pathogenic Variants and in a Cellular Model of PGRN Deficiency. *Front Mol Neurosci* **2021**, *14*, 676478,
3. Gaweda-Walerych, K.; Mohagheghi, F.; Zekanowski, C.; Buratti, E. Parkinson's disease-related gene variants influence pre-mRNA splicing processes. *Neurobiol Aging* **2016**, *47*, 127-138,
4. Chung, S.W.; Choi, B.M.; Kim, J.Y.; Lee, Y.S.; Yoon, J.P.; Oh, K.S.; Park, K.S. Altered Gene and Protein Expressions in Torn Rotator Cuff Tendon Tissues in Diabetic Patients. *Arthroscopy* **2017**, *33*, 518-526 e511,
5. Gong, C.; Kim, Y.K.; Woeller, C.F.; Tang, Y.; Maquat, L.E. SMD and NMD are competitive pathways that contribute to myogenesis: effects on PAX3 and myogenin mRNAs. *Genes Dev* **2009**, *23*, 54-66,
6. Thompson, J.C.; Stopford, C.L.; Snowden, J.S.; Neary, D. Qualitative neuropsychological performance characteristics in frontotemporal dementia and Alzheimer's disease. *J Neurol Neurosurg Psychiatry* **2005**, *76*, 920-927,
7. Desmet, F.O.; Hamroun, D.; Lalande, M.; Collod-Beroud, G.; Claustres, M.; Beroud, C. Human Splicing Finder: an online bioinformatics tool to predict splicing signals. *Nucleic Acids Res* **2009**, *37*, e67,
